# Hydrogen Peroxide‐Treated Carbon Dot Phosphor with a Bathochromic‐Shifted, Aggregation‐Enhanced Emission for Light‐Emitting Devices and Visible Light Communication

**DOI:** 10.1002/advs.201800369

**Published:** 2018-06-13

**Authors:** Zhengjie Zhou, Pengfei Tian, Xiaoyan Liu, Shiliang Mei, Ding Zhou, Di Li, Pengtao Jing, Wanlu Zhang, Ruiqian Guo, Songnan Qu, Andrey L. Rogach

**Affiliations:** ^1^ State Key Laboratory of Luminescence and Applications Changchun Institute of Optics Fine Mechanics and Physics Chinese Academy of Sciences Changchun 130033 P. R. China; ^2^ University of Chinese Academy of Sciences Beijing 100049 P. R. China; ^3^ Engineering Research Center of Advanced Lighting Technology Ministry of Education Institute for Electric Light Sources Fudan University Shanghai 200433 P. R. China; ^4^ Department of Materials Science and Engineering, and Centre for Functional Photonics City University of Hong Kong Kowloon Hong Kong SAR P. R. China

**Keywords:** aggregation‐enhanced emission, carbon dots, hydrogen peroxide, visible light communication, white‐light sources

## Abstract

It is demonstrated that treatment of blue‐emissive carbon dots (CDs) with aqueous hydrogen peroxide (H_2_O_2_) results in the green emissive solid state CD phosphor with photoluminescence quantum yield of 25% and short luminescence lifetime of 6 ns. The bathochromic‐shifted, enhanced green emission of H_2_O_2_‐treated CDs in the powder is ascribed to surface state changes occurring in the aggregated material. Using the green emissive H_2_O_2_‐treated CD phosphor, down‐conversion white‐light‐emitting devices with cool, pure, and warm white light are fabricated. Moreover, using the green emissive CD phosphor as a color converter, a laser‐based white‐light source is realized, and visible light communication with a high modulation bandwidth of up to 285 MHz and data transmission rate of ≈435 Mbps is demonstrated.

With the increasing demand for fast wireless data communication, there is a tremendous pressure on the existing broadband radio frequency (RF)/microwave wireless technologies, which are limited by bandwidth and spectrum congestion. The emerging field of visible light communication (VLC), which uses light‐emitting diodes (LEDs) or lasers for both illumination and wireless data transmission, may provide a valuable alternative approach.^1^ Compared with RF communication (including Wi‐Fi and Bluetooth), VLC has several advantages, such as higher energy efficiency, unregulated communication spectrum, environmental friendliness, higher security, and no RF interference.^2^ At the same time, commercial phosphors commonly used in white LEDs (WLEDs) are rare‐earth‐based materials, which are rather limited in supply and have characteristic luminescence lifetimes on the order of microseconds. Due to their long luminescence lifetime, intrinsic system bandwidth of rare‐earth phosphor‐based WLEDs is limited to a few MHz, which is the main obstacle for the development of high‐speed VLC.^3^ Organic semiconductors and more recently lead halide perovskite nanocrystals were suggested as color converters for VLC^4^; however, they have disadvantages of poor stability or the inclusion of toxic lead. Thus, it is of great importance to develop VLC phosphors based on abundant, nontoxic materials, with high photoluminescence quantum yield (PLQY) and short luminescence lifetimes.

Luminescent carbon dots (CDs) have recently emerged as light‐emitting nanomaterials with a high PLQY, high photostability, excellent biocompatibility, and low toxicity, which can be easily produced by both top‐down and bottom‐up chemical synthesis methods with low cost;^5^ all this endow them distinct benefits for bioimaging^6^ and lighting applications.^7^ Compared with rare‐earth phosphors, CDs have much shorter luminescence lifetimes of just a few nanoseconds, which make them potential candidate as color converter for high modulation bandwidth in VLC. However, the development of CDs for solid‐state lighting applications is greatly hindered by the fact that they often experience aggregation‐induced luminescence quenching.^8^ Continuing efforts have been paid to overcome this issue, by embedding CDs into suitable solid‐state matrix, such as polymers, inorganic salts, starch, silica, and so on.^9^ In such composite systems, high PLQYs are usually achieved at a low loading concentration of CDs, while at higher loading concentrations or in CD‐based powdered phosphors, the aggregation‐induced luminescence quenching of CDs still persists. To the best of our knowledge, there were only a few reports which introduced self‐quenching‐resistant CDs powders.^10^ For WLED and VLC applications, it is highly desirable to develop new routes leading to strongly emissive powdered CD phosphors.

In our previous studies, we found that the existence of inhomogeneous surface‐confined charges on CDs leads to the luminescence quenching in their aggregates.^11^ A proper surface engineering of CDs can help to conquer such aggregation‐induced luminescence quenching. Herein, we introduce a straightforward surface treatment method of blue‐emissive CDs with aqueous hydrogen peroxide (H_2_O_2_) solution to realize CD phosphor with an intense green emission in aggregated state (PLQY up to 25%). Utilizing this CD phosphor, WLEDs with Commission Internationale de L'Eclairage (CIE) coordinates, color temperature (CT), and color rendering index (CRI) of (0.33, 0.34), 5129 K, and 79, respectively, were fabricated. Furthermore, using the green emissive CDs phosphor as a color converter, VLC with high modulation bandwidth to 285 MHz and data transmission rate of 435 Mbps have been realized for the first time. This study provides a new method of overcoming the issue of the aggregation‐induced luminescence quenching of CDs, and demonstrates a promising utilization of CDs as an efficient phosphor for solid state lighting and, most importantly, VLC applications.

Blue‐emissive CDs were synthesized according to our previous work.^12^ Citric acid (3 g) was dissolved in 20 mL ammonia water (25% by mass) to form a transparent solution. The solution was heated in a domestic 650 W microwave oven for 5 min, resulting in formation of a dark‐brown viscous product, which was dissolved in water and centrifuged at 15 000 rpm for 10 min to remove large‐sized nanoparticles. The supernatant was freeze‐dried, dissolved in ethanol, and centrifuged at 8000 rpm for 10 min. The precipitate was collected and freeze‐dried to isolate blue‐emissive CDs, which we either denote as “original CDs” or simply as “CDs” further on. These CDs were subjected to H_2_O_2_ treatment, which included the dissolution of 1 g of CDs in 20 mL of H_2_O_2_ (6 wt% in water), followed by heating at 70 °C for 2 h, upon which the color of the solution gradually changed from opaque brown to transparent yellow. The resulting solution was concentrated in a rotary evaporator down to 3 mL, mixed with 30 mL of ethanol under vigorous stirring, centrifuged at 10 000 rpm for 10 min, and the precipitate was collected and freeze‐dried to obtain “hydrogen‐peroxide‐treated CDs,” which we will denote as “ox‐CDs” further in text.

Both CDs and ox‐CDs are easily soluble in water; their aqueous solutions show blue emission under a UV‐lamp, as can be seen in the inset of **Figure** **1**a. The absorption spectra of CDs and ox‐CDs are shown in Figure 1a. CDs exhibit the major absorption band peaked at 336 nm with a shoulder band centered at 450 nm and an absorption tail extending toward visible region. The major absorption band peaked at 336 nm can be assigned to the π–π* transitions in the carbon cores.^13^ In the ox‐CDs, the absorption below 300 nm is greatly enhanced; the major absorption peak is reduced in intensity and is bathochromic shifted toward 360 nm, while the shoulder at 450 nm and the absorption tail are nearly vanished. The enhanced absorption below 300 nm could be due to the increased amount of the oxidized functional groups on the ox‐CDs surface, while the vanished absorption at wavelengths longer than 450 nm could be attributed to passivated surface defect states as a result of the H_2_O_2_ treatment.

**Figure 1:**
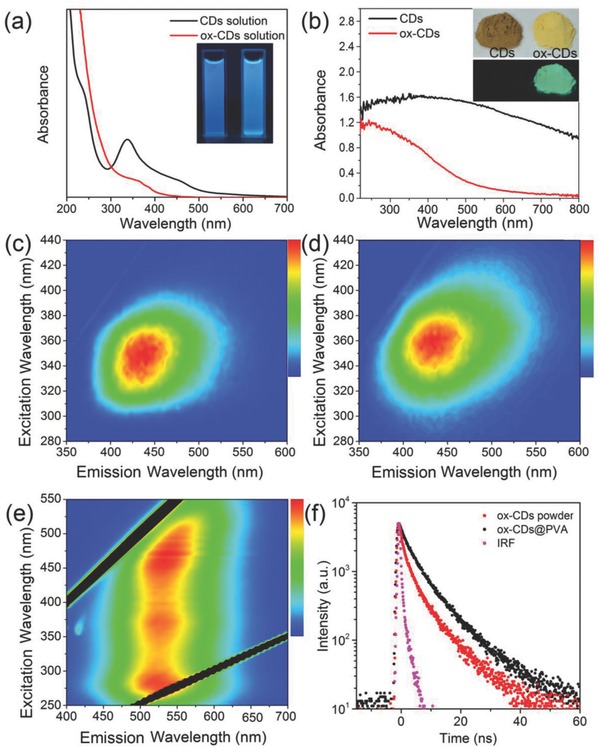
a) UV–vis absorption spectra of CDs and ox‐CDs in aqueous solutions; the inset shows photographs of these solutions under UV light. b) Diffuse reflection absorption spectra of CD and ox‐CD powders; the inset shows photographs of these powders under day light and UV light. Excitation–emission maps of c) aqueous solution of CDs, d) aqueous solution of ox‐CDs, and e) ox‐CD powder. f) PL decay curves of the ox‐CDs powder and the ox‐CDs@PVA films measured at 405 nm excitation and collected at wavelengths of respective PL peaks at 530 nm for ox‐CDs and 450 nm for ox‐CDs@PVA as indicated on the frame. IRF stays for instrumental response function.

The excitation‐emission maps of dilute aqueous solutions of CDs and ox‐CDs are shown in Figure 1c,d, respectively. Both samples exhibit an emission peak at around 435 nm with PLQYs of 21% for the CDs solution and 17% for the ox‐CDs solution. For the powdered CD samples, the color of the original CDs is dark brown, while the color of ox‐CDs is green (Figure 1b, inset). No obvious fluorescence is observed for the CD powder, while the ox‐CD powder emits green light under UV light, as shown in the inset of Figure 1b. From the excitation‐emission maps of the ox‐CD powder (Figure 1e), the PL appears to be not dependent on excitation wavelength, indicating the existence of a specific luminescent center. The emission peak remains nearly unchanged at around 520 nm for the excitation wavelengths ranging from 270 to 500 nm. The excitation spectrum of ox‐CD powder monitored at 525 nm covers a broad spectrum range from 270 to 500 nm with three peaks at 280, 370, and 455 nm (Figure 1e). In the diffuse reflection absorption spectra (Figure 1b), the CD powder exhibits a broad absorption band covering the whole visible region, while the ox‐CD powder has a much narrower absorption band with the main contribution in the UV‐to‐green spectral range. Comparing the excitation‐emission maps of the ox‐CDs in the powder (Figure 1e) and in dilute aqueous solution (Figure 1d), it can be seen that the PL peak of the powder (at 525 nm) experienced a remarkable bathochromic shift of 90 nm as compared with aqueous solution (435 nm). We note that for the ox‐CDs dispersed in a polyvinyl alcohol (PVA) matrix, the position of the PL maximum remains in the blue, peaking at 445 nm (Figure S1, Supporting Information). PL decay curves measured for the ox‐CD powder and ox‐CDs in a PVA film are shown in Figure 1f. The PL lifetime of the ox‐CD powder at 525 nm (6 ns) is shorter than that of the ox‐CDs embedded PVA film at 445 nm (8 ns), indicating the formation of other recombination channel in the powdered, aggregated state, which is faster than in solution. We note that such a PL lifetime (6 ns) is much shorter than for the rare‐earth phosphors (microseconds), offering the great potential of CDs for the VLC.

Transmission electron microscopy (TEM) images of CDs and ox‐CDs are shown in **Figure** **2**a,b, respectively; they demonstrate well‐separated nanoparticles with sizes in the range of 2–4 nm. The respective size distributions (insets of Figure 2a,b) reveal that the size of CDs remains almost unchanged after H_2_O_2_ treatment. High‐resolution TEM (HRTEM) images presented in insets of Figure 2a,b show that both CDs and ox‐CDs exhibit well‐resolved lattice fringes with a spacing of 0.21 nm, which fits with the (100) crystallographic facet of graphitic carbon and indicates their similar core structures.^14^ We can expect that H_2_O_2_ treatment would mainly affect the surface of CDs. The surface chemical structures of CDs and ox‐CDs were thus investigated by energy dispersive X‐ray spectroscopy (EDX) and X‐ray photoelectron spectroscopy (XPS). The full XPS scan (Figure 2d) shows the existence of all the constituting element of C_1s_, N_1s_, and O_1s_, with peaks at 284, 400, and 531 eV, respectively.^15^ It can be inferred that CDs become nitrogen doped due to the use of ammonia as a coreactant. As compared with the original CDs, the oxygen content in ox‐CDs is increased, which also agrees with the EDX data (Figure 2c). The high‐resolution O_1s_ XPS spectra (Figure 2f) reveal that the relative contribution of C=O/COO signal (at 531.6 eV) in ox‐CDs is much higher than in CDs.^16^ It can be inferred that the amount of carbonyl and/or carboxyl groups on the surface of ox‐CDs is increased as a result of the H_2_O_2_ treatment. The high‐resolution N_1s_ XPS spectra have three peaks at 399.4, 400.2, and 401.4 eV, corresponding to pyridinic N (C—N—C), graphite N (N—(C)_3_), and amino N (N—H), respectively (Figure 2e). The graphitic N may originate from the carbon core, while the amino‐N and pyridinic N are most probably located close or at the surface of CDs. Compared with original CDs, the N—H signal decreases and C—N—C signal increases in the ox‐CDs, indicating decreased amount of amino groups and increased amount of pyridinic N groups on the surface of ox‐CDs. Considering the electron‐donating ability of N—H group and the electron‐withdrawing feature of carbonyl, carboxyl, and pyridinic N groups, it can be inferred that the surface charge distributions of ox‐CDs tend to be more homogeneous in comparison with CDs.

**Figure 2:**
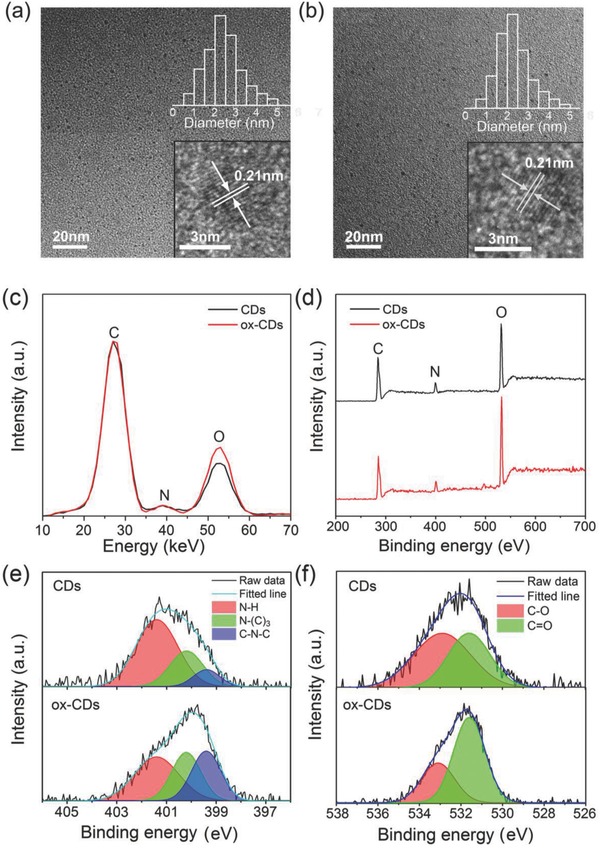
TEM images of a) CDs and b) ox‐CDs; insets show representative HRTEM images of the respective particles, and size distribution histograms. c) EDX spectra, d) full survey XPS spectra, e) N_1s_, and f) O_1s_ XPS spectra of CDs and ox‐CDs.

A possible reason for the different phenomena observed in the powders of CDs and ox‐CDs, namely, an aggregation‐induced luminescence quenching for the original CDs, and the occurrence of aggregation‐enhanced, bathochromically shifted green emission for ox‐CDs is given in **Scheme** **1**. The shoulder at 450 nm and the absorption tail in the visible region in the solution of original CDs (Figure 1a) most probably stem from the surface states, which are energetically situated within the “bandgaps” determined by the intrinsic state of the inner carbon cores. In CD aggregates, the surface states of the CDs are coupled and their “bandgaps” are further narrowed, leading to the much broadened absorption band. As often observed for aggregated luminophores (including CDs), the coupled surface states in the aggregates open additional nonradiative channels, which lead to the aggregation induced luminescence quenching.^17^ As a result of the H_2_O_2_ treatment, the surface of ox‐CDs becomes oxidized, which shifts the position of their surface state levels to higher energy; this is demonstrated by the enhanced absorption below 300 nm, decreased shoulder band at 450 nm, and the vanished absorption tail in the visible region (Figure 1a). Due to the increased amount of electron‐withdrawing group (C=O/COO) on the surface of ox‐CDs, their major absorption band which can be assigned to the π–π* transitions in the carbon cores red‐shifted to 360 nm. In aggregates, the surface states of ox‐CDs couple and as a result become situated within the “bandgap” of the carbon core, albeit with a larger energy spacing in between them. Different from the aggregated original CDs, it appears that the recombination over these coupled surface state occurs radiatively, resulting in the observed green emission. The excitation independent green emission may be induced by energy/charge transfer from the noncoupled surface states and the intrinsic states to the coupled surface states. The PLQYs of ox‐CD powders under different wavelength excitations have been estimated as 25% for excitation at 450 nm, 20% at 405 nm, and 15% at 365 nm. The gradually decreased PLQYs from lower to high excitation energy may be due to the energy losses in energy/charge transfer processes from the noncoupled surface states and the intrinsic states to the coupled surface states.

**Scheme 1:**
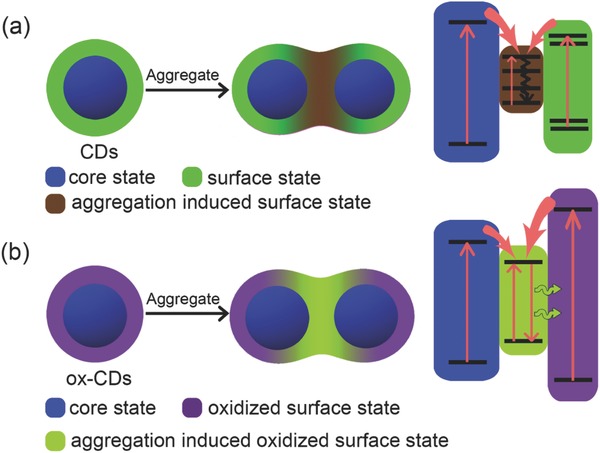
Schematic of a) CDs and b) ox‐CDs in dispersed and aggregated states; frames on the right show possible band‐energy structures and quenching processes of CDs in the aggregated state and recombination processes of ox‐CDs in the aggregated state.

Ox‐CDs powders dispersed in epoxy resin have been employed as a color down‐conversion phosphor to fabricate WLED. By deposition on the blue‐emissive (450 nm) InGaN LED chip and the adjusting of concentration of ox‐CDs powder in the color down‐conversion layer, both CIE coordinates and the CT of the resulting WLEDs can be controlled on demand, as shown in Figure S2 in the Supporting Information. By changing the mass ratio of the ox‐CDs phosphor to epoxy resin at 1: 2, 1:1, and 2: 1, WLEDs with cool white, pure white, and warm white light have been realized. **Figure** **3**a shows the emission spectra and characteristics of a representative ox‐CD‐based WLED with CIE coordinates, CT, and CRI of (0.33, 0.34), 5129 K, and 79, respectively. Figure 3b shows the photograph of pen caps with different colors under such operating WLED. The mentioned red‐shifted emission in the ox‐CD‐base LED is due to the self‐reabsorption of ox‐CD phosphors in the output emission path. This can be confirmed by comparison of the PL peak positions of two blocks of mixtures of the ox‐CD phosphors and PDMS with different mass ratios (0.5:1 and 2:1 wt%) but the same thickness (Figure S4, Supporting Information). The PL emission spectrum of the block with high mass ratio of the hybrid phosphors possesses an obvious red‐shift compared with that with the low mass ratio of the hybrid phosphors.

**Figure 3:**
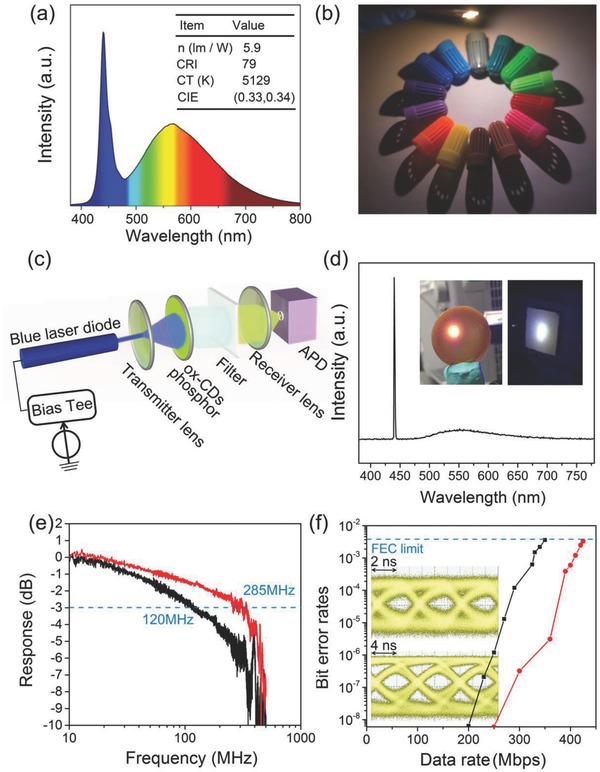
a) Emission spectrum of a down‐conversion WLED based on the ox‐CDs phosphor. The inset provides characteristics of the device. b) Photograph of pen caps with different colors illuminated with an ox‐CD‐based WLED. c) Schematic diagram of the small‐signal frequency‐response and data transmission measurements. d) Spectrum of the white light generated using blue laser diode (spike at 442 nm) and ox‐CDs phosphor. Inset: photograph of plate that dope ox‐CDs into the epoxy resin (left), and generated white light (right). e) Frequency response of the ox‐CDs (black line) and white‐light (red line) source combining the blue laser and ox‐CDs. The dotted line corresponds to the −3 dB bandwidth of the system. f) BER at different data rates using OOK of the ox‐CDs (black line) and white‐light (red line). The dash line represents the FEC threshold of 3.8 × 10^−3^. Inset: eye diagrams versus data rates of the ox‐CDs‐converted light at 150 Mbps, and white light at 300 Mbps.

In consideration of the short fluorescent lifetime of 6 ns characteristic for ox‐CDs, high modulation bandwidth and high data rate may be achieved based on this phosphor, which makes them a suitable candidate as color converter to generate white light for VLC applications. To determine the modulation bandwidth of the ox‐CDs phosphor‐converted light, small‐signal frequency‐response measurements were performed using the experimental setup shown in Figure 3c. Ox‐CDs phosphor was excited by a low‐power 450 nm blue laser diode (34.5 mA), driven by a bias‐tee combining a direct current from a Yokogawa GS610 current source with the small signal from an Agilent N5225A network analyzer (10 MHz–50 GHz). The output light was collimated by a transmitter lens and focused by a receiver lens. The intensity of the blue light from the excitation source was attenuated. The generated white light included the blue emission component from the laser diode, and the ox‐CD phosphor‐converted light (Figure 3d), whose CT could be controlled by the output blue light. The optical signal has been recorded and converted into electrical signal by an avalanche photodiode (APD430, 400 MHz). Figure 3d shows the spectrum of the generated white light with CIE coordinates of (0.34, 0.37) and CT of 5240 K. Figure 3e provides the frequency response and −3 dB modulation bandwidth characteristics of the green light from ox‐CDs and the output white light, respectively. By cutting off the blue light from the laser diode using a 495 nm long‐pass optical filter, the emission from ox‐CDs exhibits a relatively high bandwidth of 120 MHz, which is significantly larger than those of the conventional nitride‐based phosphor (≈12.4 MHz), or yttrium aluminum garnet‐based phosphor (3–12 MHz).^18^ Furthermore, the output white light exhibited a much higher bandwidth of 285 MHz. Using the ox‐CD phosphor, we further demonstrated the data transmission of phosphor‐converted VLC using a nonreturn‐to‐zero on‐off keying (NRZ‐OOK) modulation scheme. These optical pulses were detected by a high‐sensitivity APD (APD12702, 100 MHz). Figure 3f presents the bit error rate (BER) as a function of the data rate using OOK. The maximum achievable data rate of the ox‐CDs converted light and white light are 350 Mbps with a BER of 3.6 × 10^−3^, and 425 Mbps with a BER of 3.3 × 10^−3^, respectively. The BERs are both below the forward error correction (FEC) threshold required for error‐free operation. The inset of Figure 3f shows eye diagrams for the ox‐CDs phosphor‐converted light at 150 Mbps and white light at 300 Mbps. The open eyes show that the difference between zero and one bits is clearly resolved at these data rates for ox‐CDs color converter. We note that the maximum data rate of the white light is relatively slow, which is mainly ascribed to the limited bandwidth of the APD used (100 MHz). These results demonstrate the potential of the ox‐CDs as color converter in both solid‐state lighting and VLC applications. At this stage of research, the PLQY of the ox‐CD phosphor is still lower than commercial phosphors and semiconductor quantum dots. Further efforts are necessary to improve the PLQY of CD‐based phosphors to promote their practical lighting and VLC applications.

In summary, we demonstrated a convenient method of a solution treatment of blue‐emissive CDs with aqueous H_2_O_2_ to produce green emissive CD phosphor with high PLQY reaching 25% in the solid state, and short PL lifetime of 6 ns. The bathochromic‐shifted emission of the ox‐CDs in the powdered state is ascribed to the new recombination channel formed by oxidized surface states. Using the green emissive ox‐CDs phosphor, down‐conversion WLEDs with cool, pure, and warm white light were fabricated. More importantly, laser‐based lighting and data communication based on the ox‐CDs phosphor as a fast color converter were demonstrated for the first time. Ox‐CDs phosphor‐converted green light has a high modulation bandwidth (≈ 120 MHz) and high data transmission rate (using OOK ≈ 350 Mbps). The measured modulation bandwidth of ox‐CDs is more than 20 times higher than of conventional phosphors. The generated white light consisting of the 450 nm laser diode emission and ox‐CDs phosphor‐converted light exhibits CIE coordinates of (0.34, 0.37), CT of 5240 K, CRI of 79, and high modulation bandwidth of 285 MHz. The fast response and desirable color characteristics of ox‐CDs phosphor as a color converter pave the way for a new generation of dual function system for both high‐efficiency solid state lightning and high‐speed VLC.

## Conflict of Interest

The authors declare no conflict of interest.

## Supporting information

Supplementary
