# Supplementary material for: Hydrogen Peroxide‐Treated Carbon Dot Phosphor with a Bathochromic‐Shifted, Aggregation‐Enhanced Emission for Light‐Emitting Devices and Visible Light Communication
Source: Adv Sci (Weinh). 2018 Jun 13;5(8):1800369. doi: 10.1002/advs.201800369 (PMC6097149; doi:10.1002/advs.201800369)
Supplement: Supplementary file 1 — Supplementary [file ADVS-5-1800369-s001.pdf]

Copyright WILEY-VCH Verlag GmbH & Co. KGaA, 69469 Weinheim, Germany, 2018.

Supporting Information

for *Adv. Sci.,* DOI: 10.1002/advs.201800369

Hydrogen Peroxide-Treated Carbon Dot Phosphor with a Bathochromic-Shifted, Aggregation-Enhanced Emission for Light-Emitting Devices and Visible Light Communication

Zhengjie Zhou, Pengfei Tian, Xiaoyan Liu, Shiliang Mei, Ding Zhou, Di Li, Pengtao Jing, Wanlu Zhang, Ruiqian Guo, Songnan Qu,* and Andrey L. Rogach*

Supporting Information

Hydrogen Peroxide-Treated Carbon Dot Phosphor with a Bathochromic-Shifted, Aggregation-Enhanced Emission for Light-Emitting Devices and Visible Light Communication

Zhengjie Zhou, Pengfei Tian, Xiaoyan Liu, Shiliang Mei, Ding Zhou, Di Li, Pengtao Jing, Wanlu Zhang, Ruiqian Guo, Songnan Qu*, and Andrey L. Rogach*

Experimental Section

Materials: Citric acid (99.5%), NH3·H2O (≈25%) and hydrogen peroxide (≈30%) were purchased from Aladdin. PDMS elastomer kits (Sylgard 184) were purchased from Dow Corning (Midland, MI). All chemicals were used without further purification.

Fabrication of LEDs from the ox-CDs Phosphors: InGaN LED chips were purchased from Shen Zhen Hongcai Electronics CO., Ltd. The microchip, which emitted 450 nm light at the operating voltage of 3.0 V, was placed at the bottom of the LED base. For the preparation of the color conversion layer, the ox-CDs phosphors were foremost mixed with the PDMS precursors with different mass ratios of 1: 2, 1:1 and 2: 1, respectively. The mixtures were filled into the cup-shaped void of LED chip. After curing at 80 °C for 1 h, the LEDs based on ox-CDs phosphors were obtained.

Characterization: UV–visible absorption spectra were obtained on a Shimadzu UV-3101PC spectrophotometer. PL spectra were collected on a Hitachi F-7000 spectrophotometer. The PLQYs of CDs aqueous solution, ox-CDs solution, and ox-CDs powder were measured in a calibrated integrating sphere in FLS920 spectrometer under 405 nm excitation. TEM was performed on a FEI Tecnai-G2-F20 TEM at 200 kV. SEM images were obtained on a JEOL FESEM 6700F electron microscope with primary electron energy of 3 kV. Time-resolved PL spectra were measured on a LifeSpec-II dedicated lifetime spectrometer (Edinburgh Instruments). EDS and elemental mapping were conducted on an Inca X-Max instrument (Oxford Instruments). XPS was performed on a VG ESCALAB MKII spectrometer with a Mg KR excitation (1253.6 eV). The fluorescence microscopy images were obtained on a C2+ confocal microscope system (Nikon confocal instruments).

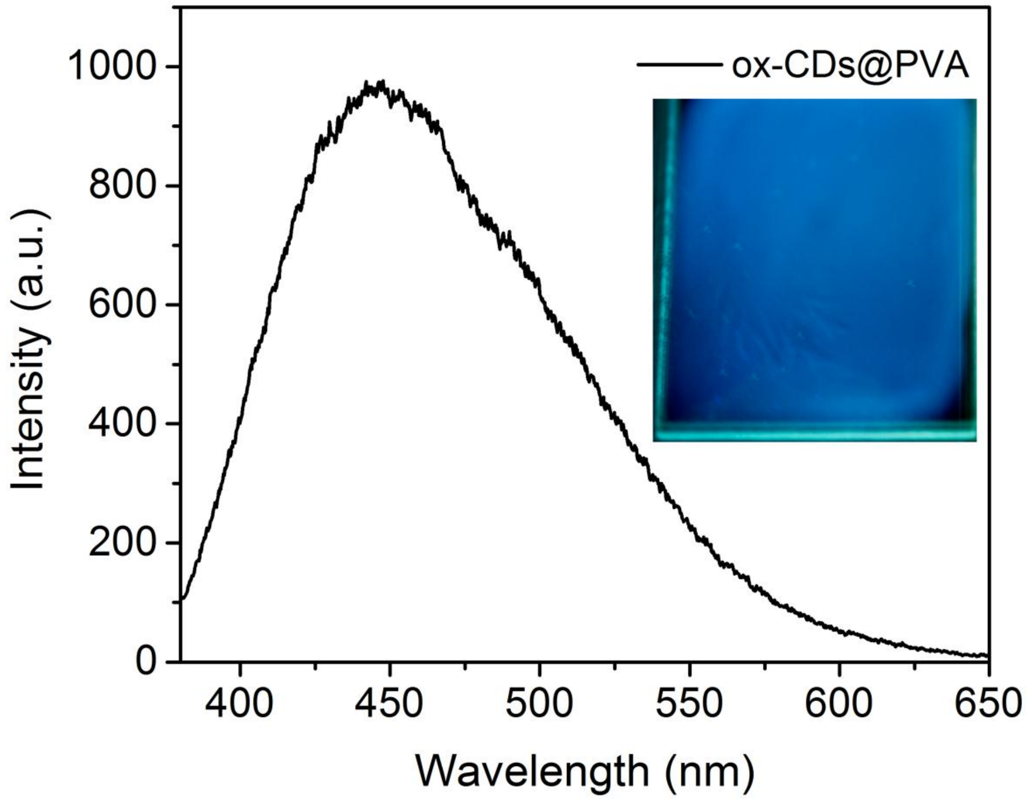

Figure S1. PL spectrum of ox-CDs@PVA film excited at 360 nm. The inset shows the photograph of the film under a UV lamp.

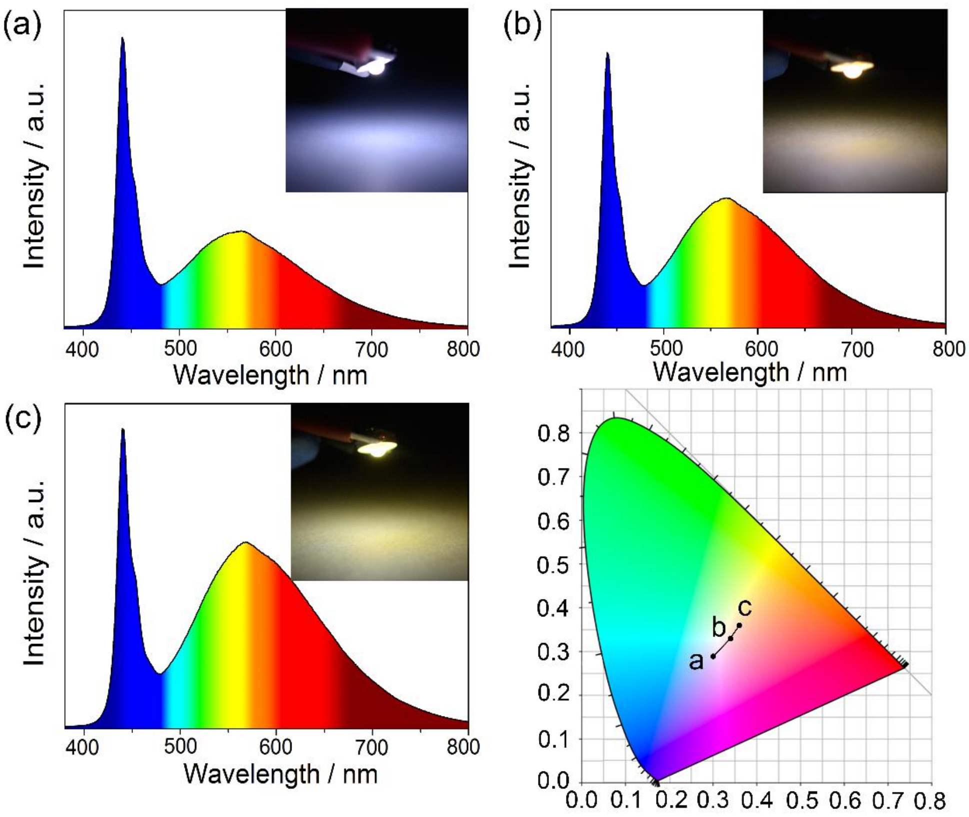

Figure S2 (a-c) Emission spectra of three ox-CD based WLEDs with different color temperatures as indicated on the frames; insets in (a-c) are photographs of the working LEDs. (d) CIE chromaticity diagram showing (x, y) color coordinates of these LEDs.

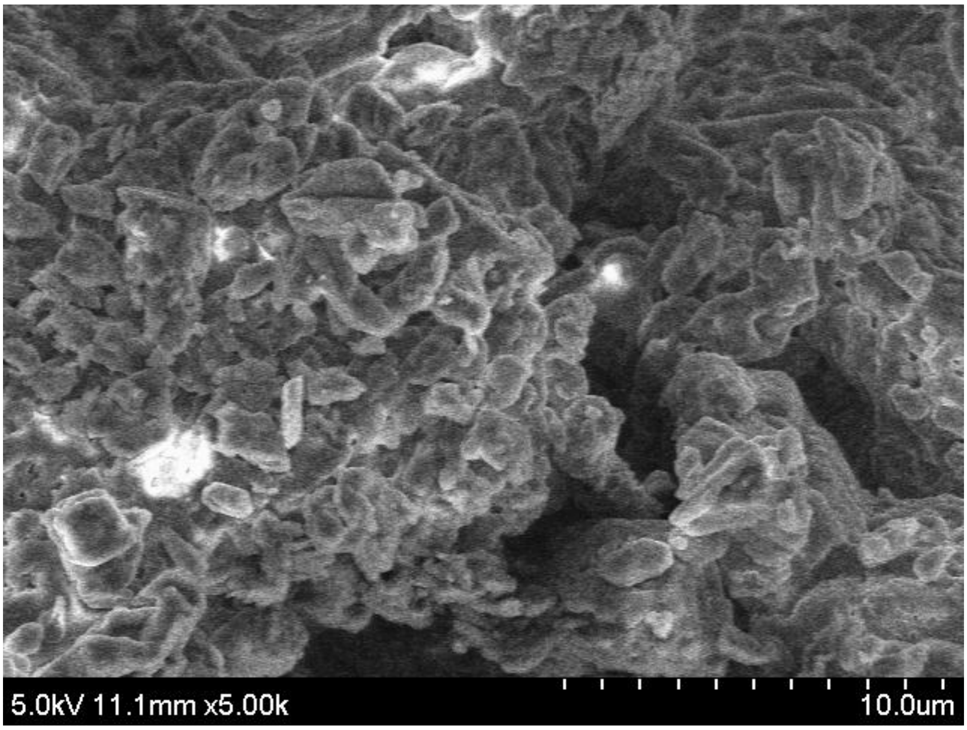

Figure S3 The SEM image of ox-CD powder.

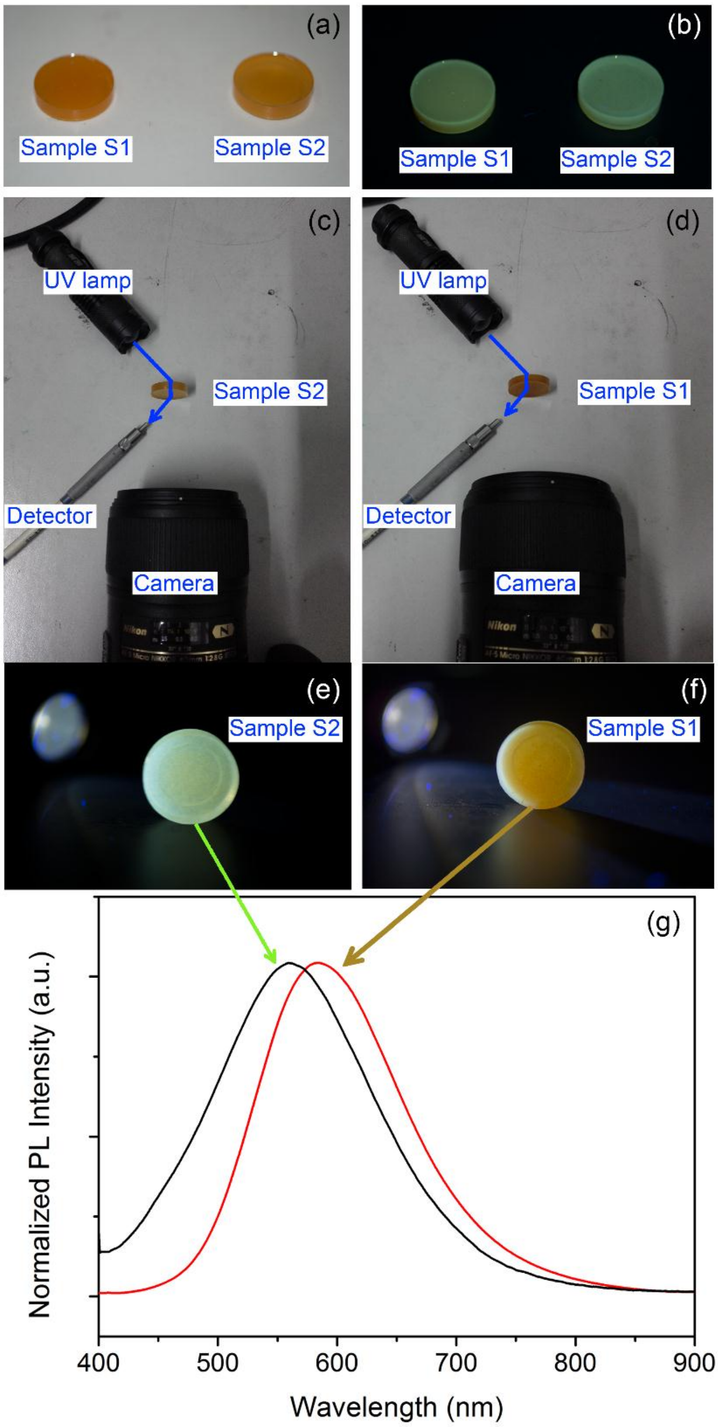

Figure S4 . Comparison of the PL peak positions of two blocks of mixtures of the ox-CD phosphors and PDMS with different mass ratios (0.5:1 and 2:1 weight %) but the same thickness. (a) Optical and (b) PL images of two blocks with low (sample S2, 0.5:1 weight %) and high (sample S1, 2:1 weight %) mass ratio. (c and d) The excitation light source and the detector are placed on the different side. (e and f) The PL images taken by the camera in (c and d). (g) The corresponding PL spectra of the two blocks.
